# Prospective Quasi-Experimental Study of Postoperative Pain Following Class II Composite Restorations Using the Snow-Plow and Resin-Coating Techniques

**DOI:** 10.3390/jcm14228107

**Published:** 2025-11-16

**Authors:** Alaa Al-Haddad, Tuleen Alwahesh, Tayma Dweikat, Dana Sharayiah, Alaa Sabrah, Rawan Elkarmi

**Affiliations:** 1 Restorative Dentistry Department, School of Dentistry, The University of Jordan, Queen Rania Street, Amman 11942, Jordan; a.sabrah@ju.edu.jo; 2 Dental Department, Jordan University Hospital, Queen Rania Street, Amman 11942, Jordan; dr.tuleenalwahesh@gmail.com (T.A.); dr.tayma1@gmail.com (T.D.); 3 Faculty of Dentistry, Al-Ahliyya Amman University, Amman 19111, Jordan; d.alsharayiah@ammanu.edu.jo; 4 Department of Pediatric Dentistry and Orthodontics, School of Dentistry, The University of Jordan, Queen Rania Street, Amman 11942, Jordan; r.elkarmi@ju.edu.jo

**Keywords:** postoperative pain, composite restorations, snow-plow technique, resin coating, immediate dentin sealing, prospective study

## Abstract

**Background/Objectives**: Postoperative sensitivity remains a common challenge following direct composite restorations, especially in Class II cavities with deep proximal boxes. The snow-plow and resin-coating techniques have been proposed to improve marginal adaptation and reduce postoperative discomfort; however, comparative clinical data remain limited. This prospective, split-mouth, quasi-experimental study aimed to compare postoperative pain associated with Class II restorations placed using either the snow-plow or resin-coating technique. **Methods**: This prospective, split-mouth study followed 83 adult patients (aged 18–45 years) who received bilateral Class II composite restorations for one week. The study received ethical approval. Each participant received one restoration using the snow-plow technique and another using the resin-coating approach. Pain intensity was evaluated using a 10-point visual analog scale (VAS) at baseline, 24-h, 72-h, and 1-week postoperatively. Analyses included Wilcoxon signed-rank, Friedman, Chi-square, McNemar, and two-way repeated-measures ANOVA tests. **Results**: Pain intensity peaked at 24-h for both techniques and declined significantly by 72-h and 1 week (*p* < 0.001). The snow-plow technique showed slightly lower mean pain scores at 24 and 72 h (*p* = 0.026 and *p* = 0.004, respectively), though categorical analyses revealed no significant difference in pain-free or minimal-pain proportions at any interval (*p* > 0.05). Both techniques showed significant within-group reductions in pain over time (*p* < 0.001). **Conclusions**: Both restorative approaches demonstrated similar postoperative pain trajectories, with substantial improvement by one week. While minor differences in early mean pain intensity were observed, these were not clinically significant. The findings suggest that either technique can be effectively employed to achieve satisfactory postoperative comfort when modern adhesive protocols are applied. Clinicians can therefore select either technique based on preference and clinical circumstances, with the expectation of comparable short-term postoperative comfort outcomes.

## 1. Introduction

Effective management of postoperative dental pain remains a significant clinical challenge across all branches of restorative dentistry. Typically, pain intensity peaks within the first 24 h following restorative procedures as a consequence of tissue injury, pulpal inflammation, or mechanical irritation, then gradually subsides over subsequent days. Inadequate pain control not only affects immediate comfort but can also discourage patients from seeking future care and impair their oral health-related quality of life [1].

Postoperative sensitivity following direct composite restorations is among the most frequently reported complications, with prevalence rates of up to 50% [2]. Key etiological factors include polymerization shrinkage stress, marginal leakage, occlusal discrepancies, and pulpal irritation [3]. Class II restorations, in particular, are susceptible to such complications due to their high configuration factor (C-factor) and deep proximal boxes, which can limit light penetration and compromise marginal integrity [4,5].

Various operative strategies have been developed to mitigate these effects. Incremental layering techniques reduce shrinkage stress, while bulk-fill composites permit deeper curing and simplify application [6]. Among adjunctive strategies, the snow-plow and resin-coating techniques have gained attention for improving marginal sealing and reducing postoperative sensitivity [7,8,9,10,11,12].

The snow-plow technique involves applying an uncured flowable composite to the gingival floor, followed by immediate placement of a packable composite increment, which displaces the flowable resin into interfacial voids and improves marginal adaptation [9,10]. Conversely, resin coating after immediate dentin sealing (IDS) involves applying an adhesive and a thin cured flowable composite layer over freshly cut dentin to form a stress-absorbing seal [11,12].

Despite laboratory and limited clinical evidence supporting both techniques, direct clinical comparisons regarding their effect on postoperative pain are scarce. To date, there is a notable lack of clinical studies directly comparing the postoperative pain profiles associated with the snow-plow and resin-coating techniques. Understanding how these approaches influence short-term patient comfort is crucial for evidence-based decision-making. This study aims to fill this knowledge gap by providing a direct clinical comparison.

Accordingly, this prospective split-mouth clinical trial was conducted to monitor and compare the pattern and intensity of postoperative pain following Class II restorations placed using either the snow-plow or resin-coating technique under standardized clinical conditions. The null hypothesis (H_0_) was that there would be no difference in postoperative pain intensity between restorations placed with the snow-plow technique and those placed with the resin-coating technique over a one-week period. The alternative hypothesis (H_1_) was that a significant difference would exist.

## 2. Materials and Methods

### 2.1. Study Design and Setting

This was a prospective, split-mouth, quasi-experimental study conducted at the Restorative Clinics of Jordan University Hospital between January and September 2025. Ethical approval was obtained from the Academic Research Committee of the University of Jordan (Ref: 2/5/1/200). All participants provided written informed consent prior to inclusion. The study adhered to the principles of the Declaration of Helsinki, and the reporting of this prospective, split-mouth, quasi-experimental study was designed to comply with the principles of transparent research reporting.

**Figure 1:**
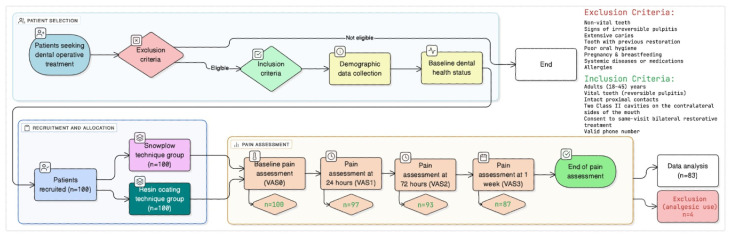
Schematic presentation of the study protocol, illustrating the total number of participants evaluated, enrolled, and subjected to analysis.

A total of 100 adult patients scheduled for bilateral Class II composite restorations were initially enrolled. Of these, 83 patients (166 restorations) completed all follow-ups and were included in the final analysis. Participants were observed for the development and intensity of postoperative pain at specific intervals following treatment (Figure 1).

### 2.2. Inclusion and Exclusion Criteria

Eligible participants were 18–45 years old, each presenting with two contralateral Class II carious lesions in vital teeth diagnosed with reversible pulpitis. Teeth required intact proximal contacts, functional occlusion, and good oral hygiene. Exclusion criteria included pregnancy, irreversible pulpitis or necrosis, deep caries approaching the pulp, prior endodontic treatment, periodontal inflammation, parafunctional habits, or recent use of analgesics or anti-inflammatories. These criteria mirror contemporary postoperative sensitivity and Class II composite trials to ensure a homogeneous, low-risk cohort for pain outcomes [13,14,15,16,17,18].

### 2.3. Clinical Procedures

All restorations were performed by the same experienced restorative specialist to maintain procedural consistency. Each patient received two restorations: one using the snow-plow technique and one using the resin-coating technique. Class II lesions and their depth extension (~0.5 mm away from pulp) were confirmed with bitewing radiographs by an assistant dentist. The assignment and sequence of techniques followed a systematic alternation pattern, with the first technique applied to the right side for the first patient, then alternating sides for subsequent patients to minimize selection bias.

#### 2.3.1. Common Protocol

Local anesthesia (2% lidocaine with 1:100,000 epinephrine) and rubber dam isolation were used. Cavities were prepared with high-speed diamond burs under continuous water cooling with new high-speed diamond burs to ensure efficient cutting, minimize heat generation, and prevent pulpal irritation, thereby reducing the risk of postoperative sensitivity [19,20]. A sectional matrix system (Palodent V3, Dentsply Sirona, Konstanz, Germany) was applied. Enamel margins were selectively etched with 37% phosphoric acid for 20 s, rinsed, and air-dried. A universal adhesive (Prime & Bond Universal, Dentsply Sirona, Konstanz, Germany) was applied to enamel and dentin, air-thinned, and light-cured for 20 s using an LED unit (output ~1400 mW/cm^2^). The adhesive layer was allowed to mature for 5 min (“decoupling with time”) to enhance hybrid layer stability as previously recommended [21].

#### 2.3.2. Resin-Coating Technique

Immediately after adhesive maturation, a thin (~0.5 mm) layer of SDR bulk-fill flowable composite (Dentsply Sirona, Konstanz, Germany) was applied to cover the cavity floor, meticulously spread and adjusted with a periodontal probe, followed by light curing for 20 s [22,23]. A packable composite (Spectrum A2, Dentsply Sirona, York, PA, USA) was used to build the proximal wall, thereby transforming the preparation into a Class I configuration. The remaining cavity was then filled with SDR bulk-fill composite in a single increment, and the occlusal morphology was completed by incrementally placing the packable composite to restore the full anatomy. Occlusion was verified with 30-µm articulating paper, and necessary adjustments were made.

#### 2.3.3. Snow-Plow Technique

After the adhesive curing and maturation, an uncured 0.5 mm layer of SDR flowable composite was applied to the gingival floor, followed by immediate placement of a packable composite increment. This allows the flowable composite to adapt to micro-irregularities and voids displaced by the packable composite [12,24]. Both were light-cured simultaneously to transfer the cavity configuration into class I. The remaining cavity was filled as described above. After rubber dam removal, occlusion was verified using articulating paper and adjusted as necessary. Final polishing was performed with fine-grit polishing stones.

#### 2.3.4. Standardization Measures

To minimize confounding variables, the clinical protocol was rigorously standardized between the two techniques. The same operator performed all procedures using identical materials and equipment. Clinical observation confirmed that both techniques required similar operative time investments, with procedures consistently completed within the standard clinical timeframe expected for Class II composite restorations of comparable complexity. All other aspects of the restorative process, including isolation, adhesive application, curing protocols, and finishing/polishing, were maintained identically between groups.

### 2.4. Pain Assessment

Postoperative pain was assessed using a 10-point visual analog scale (VAS), where 0 = no pain and 10 = worst imaginable pain. Baseline pain (VAS0) was recorded prior to treatment, followed by assessments at 24 h (VAS1), 72 h (VAS2), and 1 week (VAS3). A calibrated, blinded assessor conducted follow-up telephone interviews using a standardized script (see Supplementary File). The assessor was blinded to the technique assigned to each specific tooth throughout the follow-up period. Patients were instructed to report pain for each tooth independently. Analgesic intake (ibuprofen 400 mg as needed) was recorded if used. This approach has been validated in previous studies evaluating postoperative dental pain [18,25,26]. Prior to the commencement of the study, the assessor received training in the application of the Visual Analogue Scale (VAS). Intra-examiner reliability for pain scoring was verified during calibration sessions involving non-study patients, demonstrating high reliability (Cohen’s κ = 0.80) [27]. Patients were not informed of the specific technique applied to each tooth in order to minimize expectation bias, and the assessor was blinded to the allocation of techniques to prevent observer bias [28].

### 2.5. Statistical Analysis

Sample size was determined a priori using G*Power (version 3.1, University of Düsseldorf, Germany): for a two-tailed Wilcoxon signed-rank test, assuming a clinically relevant paired mean difference of 1.0 VAS unit, SD of paired differences ≈2.0 (dz ≈ 0.5), α = 0.05, and 80% power, 34 pairs were required; to accommodate non-normal analyses, repeated measures, and attrition, a target of ≥80 patients was set, and 100 were recruited. Data were analyzed using IBM SPSS Statistics (version 26, IBM Corp., Armonk, NY, USA). Because VAS scores were non-normally distributed (Shapiro–Wilk, *p* < 0.05), non-parametric analyses were used. Continuous data are presented as mean ± SD, and categorical data (No pain: 0; Mild: 1–3; Moderate: 4–6; Severe: 7–10) as frequencies and percentages.

Comparisons between techniques at each time point were made using the Wilcoxon signed-rank test. Changes over time were evaluated using the Friedman test with Bonferroni correction. Proportions of pain-free (VAS = 0) and minimal-pain (VAS ≤ 1) outcomes were compared using McNemar and Cochran’s Q tests. Two-way repeated-measures ANOVA examined time × technique interaction effects. Despite non-normal data distribution, this parametric test was appropriate for its robustness and the split-mouth design’s power. Significance was set at *p* < 0.05 unless adjusted.

## 3. Results

### 3.1. Participant Characteristics

Eighty-three participants (51 females, 32 males; mean age 28.4 ± 8.2 years) completed all assessments, contributing 166 restorations (83 per technique). Demographic and baseline characteristics are summarized in Table 1.

### 3.2. Postoperative Pain Intensity

**Figure 2:**
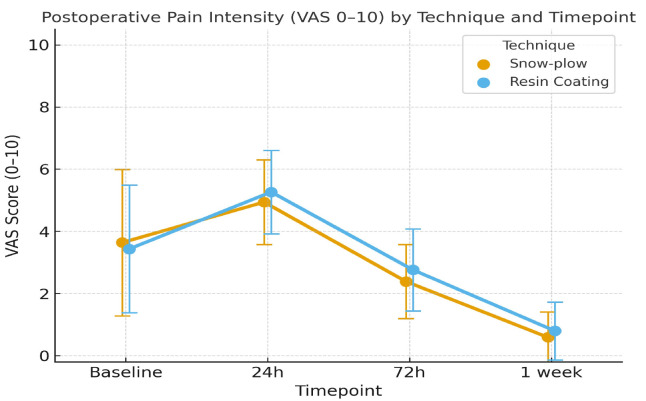
Mean Postoperative Pain Intensity (VAS 0 –10) Over Time for Snow-Plow and Resin Coating Techniques. (Note: Error bars represent standard deviations.

Mean pain scores for each technique and time point are summarized in Table 2. Pain peaked at 24 h for both methods, with progressive decline at 72 h and 1 week (*p* < 0.001; Figure 2).

Wilcoxon signed-rank tests revealed no baseline difference between techniques (*p* = 0.44). The snow-plow technique demonstrated significantly lower mean pain at 24 h (*p* = 0.026) and 72 h (*p* = 0.004), but not at 1 week (*p* = 0.053). Friedman tests confirmed significant temporal changes within each technique for both methods (*p* < 0.001). A two-way repeated-measures ANOVA revealed a significant main effect of time (*p* < 0.001), whereas the main effect of techniques was not statistically significant (*p* = 0.114). Despite statistical significance in the Wilcoxon tests, the effect sizes for the differences at 24 and 72 h were small (r = 0.25 and r = 0.33, respectively), supporting the lack of clinical relevance. Additionally, the analysis indicated a significant interaction between time and technique (*p* = 0.003), suggesting divergent pain trajectories despite similar overall results. Exploratory subgroup analyses based on tooth type (molar/premolar) and patient gender revealed no significant interactions or confounding effects on the primary pain outcomes (*p* > 0.05).

### 3.3. Categorical Analysis

Distributions of pain intensity categories (no pain, mild, moderate, severe) are presented in Table 3. No significant differences between techniques were found at any time point (*p* > 0.05). At 24 h, no restorations were pain-free in either group. By 72 h, 8.4% (snow-plow) and 7.2% (resin-coating) were pain-free; by one week, this increased to 60.2% and 51.8%, respectively (*p* = 0.23). Both techniques showed significant within-group increases in pain-free proportions over time (*p* < 0.001). Similarly, the percentage of restorations with VAS ≤ 1 increased to 83.1% (snow-plow) and 72.3% (resin-coating) by one week, with no statistical difference (*p* = 0.108) (Table 4).

## 4. Discussion

This prospective split-mouth trial evaluated postoperative pain outcomes following Class II composite restorations placed using either the snow-plow or resin-coating techniques. Both approaches resulted in similar pain trajectories and clinically comparable outcomes over one week. The primary finding is that while minor statistical differences in mean VAS scores were observed at 24 and 72 h, there was no clinically meaningful difference in pain experience between the snow-plow and resin-coating techniques at any time point. Therefore, the null hypothesis of no difference between techniques was accepted for the primary clinical outcome.

The temporal pattern of postoperative pain was pronounced and consistent for both techniques. Pain intensity peaked sharply at 24 h before progressively subsiding at 72 h and 1 week. This trajectory aligns with established evidence that postoperative sensitivity following posterior composite restorations typically peaks within the first 24–48 h before gradually resolving [13,14]. The categorical data revealed a particularly striking finding: no patients in either group were completely pain-free at 24 h, underscoring the universal challenge of early postoperative sensitivity in Class II restorations. However, comfort improved substantially over time, with over 50% of patients pain-free and over 70% experiencing minimal or no pain (VAS ≤ 1) by one week. These findings confirm that when contemporary adhesive protocols are rigorously followed, postoperative discomfort, while common initially, is largely self-limiting and shows substantial improvement within the first week.

The interpretation of between-technique differences requires careful consideration of the outcome measure. Analysis of continuous VAS scores showed statistically lower mean pain with the snow-plow technique at 24 and 72 h. The snow-plow technique—where an uncured flowable composite is displaced by a packable increment—theoretically improves marginal adaptation by penetrating micro-irregularities [5,9,10], which could explain this subtle difference in mean scores. However, this statistical difference was not translated into clinically meaningful categorical outcomes. Both techniques showed virtually identical rates of pain-free patients at all time points, and similar proportions of patients achieving minimal discomfort (VAS ≤ 1). This suggests that while the snow-plow technique may offer a minor statistical advantage in pain reduction, this difference does not manifest as a clinically superior ability to prevent pain or provide comfort in the early postoperative period.

Both techniques demonstrated a significant and substantial reduction in pain over time, affirming the effectiveness of modern adhesive strategies. The resin coating technique, which provides a stress-absorbing layer and protects the dentin, resulted in a marked improvement in comfort from the 24-h peak, consistent with its proposed mechanism of enhancing hybrid layer stability and acting as an elastic buffer [11,12]. Contrary to our initial expectations and some previous reports [5], the snow-plow technique did not confer a decisive clinical advantage in the early post-operative period. While laboratory studies have suggested that the snow-plow technique can improve marginal adaptation [9,10], this in vitro benefit did not translate into a clinically superior pain reduction profile in our study. The findings of Alghauli et al. [11], which highlighted the benefits of resin coating, are thus supported by our results in the context of direct restorations. Ultimately, both techniques proved to be clinically viable and effective strategies, with the choice between them likely depending more on operator preference and specific clinical circumstances than on a significant difference in post-operative comfort.

The categorical analyses provided the most clinically relevant perspective. They revealed that the experience of post-operative pain was almost universal at 24 h, with 0% of patients in either group being completely pain-free. This underscores the significant inflammatory challenge posed by Class II cavity preparations, even with optimal technique. The subsequent increase in pain-free rates, while substantial, was more gradual than often assumed, reaching only 51.8–60.2% at one week. However, the analysis of “no or minimal pain” (VAS ≤ 1) paints a more positive picture of patient comfort, showing that over 72% of patients experienced only minor discomfort by the one-week mark. Critically, none of the categorical analyses—whether for pain-free status or minimal pain—showed any statistically significant difference between the two restorative techniques at any time point. This consistently supports the conclusion of clinical equivalence.

Both techniques demonstrated comparable effectiveness in managing postoperative discomfort, with patients showing substantial improvement in comfort by one week. This outcome underscores the importance of applying evidence-based clinical protocols to minimize postoperative sensitivity. In the present study, several best practices were standardized across groups: selective enamel etching, application of a universal adhesive, adequate adhesive polymerization time, use of bulk-fill composites, C-factor reduction, strict rubber dam isolation, and meticulous finishing. Each of these steps has been shown to improve marginal adaptation, reduce polymerization stress, and enhance adhesive performance [4,5,6,7,11,12,19,20,21].

The trajectory of pain resolution observed—while more gradual than previously assumed—nevertheless confirms the self-limiting nature of postoperative sensitivity when evidence-based protocols are followed. The biological basis for this pain likely involves a combination of polymerization shrinkage stress transmitted to the pulp-dentin complex, fluid flow dynamics in the dentinal tubules, and localized inflammatory response. The resin-coating technique aims to create a stress-absorbing, sealed layer that mitigates these factors, while the snow-plow technique seeks to achieve a similar outcome by improving marginal adaptation. Our results suggest that both mechanisms are equally effective in controlling clinical pain symptoms in the short term. The finding that over 70% of patients experienced only minimal or no pain (VAS ≤ 1) by one week provides clinically meaningful reassurance. Although early pain was nearly universal, the consistent improvement across both techniques highlights that contemporary adhesive protocols can effectively manage postoperative discomfort. These findings emphasize that postoperative sensitivity can be controlled through rigorous technique, including proper isolation, optimized adhesive procedures, and stress-reducing restorative approaches.

These observations are consistent with contemporary clinical evidence on adhesive strategies and restorative techniques. Randomized controlled trials have shown that modern bulk-fill protocols, when used with appropriate bonding procedures, are associated with acceptable postoperative sensitivity profiles in Class I and II restorations [17]. The clinical equivalence observed in our study aligns with evidence that bulk-fill restorations perform as well as, or better than, incremental layering techniques with respect to patient comfort [6]. The performance of both techniques also supports the clinical versatility of contemporary composite systems, as demonstrated by studies showing the effectiveness of various bulk-fill approaches [22,29].

Laboratory evidence supports the proposed mechanistic advantages of both techniques: the snow-plow approach may improve marginal adaptation and reduce microleakage [5,9,10,30], while resin coating strategies enhance dentin protection and bond stability [11,12]. However, our clinical results suggest that these different mechanisms ultimately produce comparable postoperative comfort outcomes. Long-term follow-up studies confirm that bulk-fill restorations—including techniques similar to those examined here—maintain excellent clinical performance over several years [31,32]. Collectively, these findings align the present clinical results with evidence indicating that when sound adhesive and restorative protocols are followed, contemporary composite techniques are clinically effective, with technique selection based on factors beyond early postoperative pain.

This study possesses several key methodological strengths. The split-mouth design effectively minimized inter-individual variability, enabling a direct and powerful comparison of the two restorative approaches. Strict inclusion criteria ensured a homogeneous patient cohort, while operator consistency and standardized protocols reduced procedural variability. The use of a single calibrated and blinded assessor for pain evaluation minimized observer bias. Furthermore, the low dropout rate and the detailed analysis of both continuous and categorical pain outcomes enhance the reliability and clinical relevance of the findings.

Nevertheless, several limitations must be acknowledged. The one-week evaluation period was exclusively focused on short-term postoperative sensitivity; while adequate to capture the critical initial pain trajectory, it does not provide insights into long-term outcomes such as marginal integrity or the longevity of restorations. The study cohort comprised young adults diagnosed with reversible pulpitis, which may limit the applicability of findings to older populations or teeth presenting with deeper lesions. Furthermore, although the split-mouth design effectively controlled for inter-individual variability, the allocation of techniques followed a systematic alternation pattern rather than randomized assignment. While this method ensured a balanced distribution and minimized selection bias through predetermined alternation, it does not offer the same methodological rigor as true randomization. Nonetheless, considering that both techniques were implemented within the same oral environment for each patient, and acknowledging the clinical practicality of the alternation strategy, we assert that this approach maintained adequate internal validity for the comparative evaluation of the two restorative techniques. Additionally, although cavity preparation adhered to standardized principles, the depth of the cavities was not consistently measured during the procedures, potentially introducing variability in pulpal response. Moreover, while operative duration appeared clinically comparable between techniques, it was not precisely timed with a stopwatch nor subjected to statistical analysis; although unlikely to substantially influence the outcomes, the potential for minor, unquantified differences in operative time remains. Finally, reliance on subjective VAS scores, although validated, inherently depends on patient perception.

These limitations chart a course for future research. Long-term follow-up is essential to determine if the early pain profile observed here has any correlation with long-term clinical performance. Future studies would benefit from incorporating objective measures, such as optical coherence tomography for interfacial evaluation [33,34], thermographic monitoring of pulp vascular responses [35], modern pulp vitality testing technologies [36], or quantitative sensory testing, to complement patient-reported outcomes. Given the clinical equivalence found in this study, the exploration of hybrid techniques or the application of these strategies in more complex clinical scenarios, such as deep cavities, with standardized cavity depth measurements, could reveal specific indications where one technique may be preferred. Finally, exploring the impact of pre-operative analgesics or the development of enhanced adhesive protocols specifically aimed at mitigating the pronounced 24-h pain response represents a critical avenue for improving patient comfort.

## 5. Conclusions

Both the snow-plow and resin coating techniques resulted in similar postoperative pain profiles over one week. While early postoperative pain was substantial and nearly universal at 24 h, both techniques showed comparable and significant improvement in patient comfort over time, with no clinically meaningful differences between them. The choice between these techniques can therefore be based on operator preference, specific clinical circumstances, or long-term performance considerations rather than expected differences in early postoperative comfort. These findings provide clinicians with evidence that both techniques are viable options for managing short-term postoperative comfort in Class II restorations. However, the conclusions are applicable to the one-week period, and long-term follow-up studies are necessary to confirm the clinical stability and performance of these restorations over time.

## Figures and Tables

**Table 1:** Demographic and Clinical Characteristics of Study Cohort (*n* = 83).

**Age (Years), Mean ± SD**	**28.4 ± 8.2**
Age (Range)	18–45
Age (Median, IQR)	26 (22–33.5)
**Characteristic**	**n (%)**
Female	51 (61.4)
Male	32 (38.6)
Molar	97 (58.4)
Premolar	69 (41.4)
Quadrant: Upper Left (UL)	52 (31.3)
Quadrant: Upper Right (UR)	47 (28.3)
Quadrant: Lower Right (LR)	36 (21.7)
Quadrant: Lower Left (LL)	31 (18.7)
Technique: Resin Coating	83 (50)
Technique: Snow-plow	83 (50)

**Table 2:** Postoperative Pain Outcomes by Technique and Time Point (Mean ± SD, [95% CI]), with the Wilcoxon Signed-Rank Test Results.

Timepoint	Snow-Plow (Mean ± SD, 95% CI)	Resin Coating (Mean ± SD, 95% CI)	Wilcoxon Signed-Rank Test (*p*-Value)
Baseline (VAS_0_)	3.64 ± 2.37 [3.13, 4.15]	3.43 ± 2.06 [2.99, 3.87]	536 (0.440)
24 h (VAS_1_)	4.94 ± 1.37 [4.65, 5.23]	5.27 ± 1.35 [4.98, 5.56]	799 (0.026) *
72 h (VAS_2_)	2.39 ± 1.2 [2.13, 2.65]	2.76 ± 1.33 [2.47, 3.05]	426 (0.004) *
1 week (VAS_3_)	0.59 ± 0.83 [0.41, 0.77]	0.80 ± 0.93 [0.60, 1.00]	321 (0.053)

VAS: Visual Analogue Scale (0 = no pain, 10 = worst imaginable pain); SD: Standard Deviation; CI: Confidence Intervals. *: Statistically significant (*p* < 0.05).

**Table 3:** Distribution of Patients Across Postoperative Pain Categories [n (%)] for Snow-Plow and Resin-Coating Techniques at Various Time Points.

Time Point	Technique	No Pain (0)	Mild (1–3)	Moderate (4–6)	Severe (7–10)	χ^2^ (*p*, Within Group)	*p* (McNemar Pain-Free)
**Baseline**	Snow-plow	14 (16.9%)	49 (59.0%)	16 (19.3%)	4 (4.8%)	χ^2^ = 58.4 (*p* < 0.001)	0.688
	Resin Coating	12 (14.5%)	52 (62.7%)	17 (20.5%)	2 (2.4%)	χ^2^ = 60.1 (*p* < 0.001)	
**24 h**	Snow-plow	0 (0.0%)	13 (15.7%)	57 (68.7%)	13 (15.7%)	χ^2^ = 77.5 (*p* < 0.001)	1.000
	Resin Coating	0 (0.0%)	8 (9.6%)	58 (69.9%)	17 (20.5%)	χ^2^ = 80.9 (*p* < 0.001)	
**72 h**	Snow-plow	7 (8.4%)	63 (75.9%)	13 (15.7%)	–	χ^2^ = 66.3 (*p* < 0.001)	1.000
	Resin Coating	6 (7.2%)	53 (63.9%)	24 (28.9%)	–	χ^2^ = 68.7 (*p* < 0.001)	
**1 week**	Snow-plow	50 (60.2%)	33 (39.8%)	–	–	χ^2^ = 17.2 (*p* < 0.001)	0.230
	Resin Coating	43 (51.8%)	40 (48.2%)	–	–	χ^2^ = 20.9 (*p* < 0.001)	

Cochran’s Q (pain-free over time): Snow-plow Q(3) = 102.99 (*p* < 0.001); Resin Coating Q(3) = 88.49 (*p* < 0.001). Chi-square (χ^2^) tests compare within-technique distributions of pain severity across categories; McNemar’s test compares paired pain-free proportions between techniques at each time point. Cochran’s Q test evaluates overall changes in pain-free proportions across follow-up intervals for each technique. (*p* < 0.05 is considered significant).

**Table 4:** Proportion of Patients with No or Minimal Postoperative Pain (VAS ≤ 1).

Time Point	Snow-Plow, n (%)	Resin Coating, n (%)	*p*-Value (McNemar)
Baseline	18 (21.7%)	17 (20.5%)	1.000
24 h	0 (0.0%)	0 (0.0%)	1.000
72 h	18 (21.7%)	12 (14.5%)	0.146
1 week	69 (83.1%)	60 (72.3%)	0.108

VAS ≤ 1 was considered indicative of no or minimal postoperative pain. Values represent the number and percentage of restorations per technique at each time point. Between-technique comparisons of paired proportions were performed using McNemar’s test (exact, two-tailed; *p* < 0.05 considered statistically significant.

## Data Availability

The data presented in this study are available on request from the corresponding author. The data are not publicly available due to privacy reasons.

## Supplementary Materials

The following supporting information can be downloaded at: https://www.mdpi.com/article/10.3390/jcm14228107/s1, the structured telephone questionnaire employed, and the blinding data collection protocol.

## Abbreviations

The following abbreviations are used in this manuscript:

IDS	Immediate Dentin Sealing
VAS	Visual Analogue Scale
SD	Standard Deviation
SDR	Smart Dentin Replacement
