# Supplementary material for: Prospective Quasi-Experimental Study of Postoperative Pain Following Class II Composite Restorations Using the Snow-Plow and Resin-Coating Techniques
Source: J Clin Med. 2025 Nov 16;14(22):8107. doi: 10.3390/jcm14228107 (PMC12653662; doi:10.3390/jcm14228107)
Supplement: Supplementary file 1 [file jcm-14-08107-s001.zip › jcm-3981672-supplementary.pdf]

Supplementary File

This supplementary file includes information concerning the detailed statistical analysis conducted (Tables S1), the pain assessment sheet utilized during the telephone interview, the structured interview script followed by the assessor, and the procedures for blinded data entry and analysis.

Pain Assessment Sheet

Patient Details:

**Treatment Date:**	
**Patient ID:**	
Contact Number	
Age:	
Sex:	
Tooth Number Restorted:	

Pain Assessment:

	VAS Score		
**Time Intervals**	**Right Side:**<br>Upper ____ Lower	**Left Side:**<br>Upper ____ Lower	**Analgisc Taken**<br>Yes / NO
24 hours (VAS_1)			
72 hours (VAS_2)			
1 week (VAS_3)			

Additional Notes:

Structured Telephone Interview Form

This structured telephone interview form was employed by the blinded assessor to record postoperative pain following Class II composite restorations. Patients were contacted at intervals of 24 hours, 72 hours, and one week subsequent to the procedure. Each patient was instructed to independently report pain intensity for the restored tooth in the respective quadrant, utilizing a 0–10 Visual Analog Scale (VAS), where 0 signifies no pain and 10 indicates the maximum conceivable pain.

Blinded Post-operative Telephone Interview Script (Translated)

Pre-call instructions for assessor:

- Identify the patient by their ID.
- Identify the call window: 24 hours, 72 hours, or 1 week.
- Identify the treated teeth for the patient by quadrant, not by technique.
- Alternate the order of questioning per side based on patient IDs (even IDs commence with the right side; odd IDs commence with the left side).

Structured Script:

"Hello, may I please speak with [**Patient Name**]?"

"Hi, this is [Assessor Name] calling from the Jordan University Hospital Restorative Clinic regarding your recent dental treatment. This will take about 2–3 minutes. Is now a good time?"

If Yes,

"Great—just a reminder: I don't know which specific technique was used on each tooth. I'll ask about each treated side separately. Please answer each question for the specific tooth I mention, and try not to compare the two sides to each other when giving your answers."

"To rate any pain, please use a 0–10 scale, where 0 means no pain at all and 10 means the worst pain you can imagine."

"First, I'll ask about your [**upper right tooth].** On a 0–10 scale, what is your current pain?" (*Record score on Pain Assessment Sheet*)

"Ok, now I'll ask about your [**lower left tooth].** On a 0–10 scale, what is your current pain?" (*Record score on Pain Assessment Sheet*)

"Since the treatment, have you taken any painkillers (such as ibuprofen, Panadol, or others) for pain in either treated tooth?"

If Yes,

" Which medications did you take, when did you take them, and for which side?

(Record details within the analgesic column (Yes/No), and medication in additional notes)

"Thank you. We'll call you again at **[next timepoint**]. If you develop severe pain, swelling, or biting discomfort, don't hesitate to get in touch with the clinic using the number we provided."

Data Entry and Analysis:

Data entry and analysis were conducted sequentially and blindly according to a standardized order. Initially, the assistant dentist documented the following data for all recruited patients in an Excel spreadsheet, employing two rows:

Patient_ID, Mobile_Number, Age, Sex, Treated_Tooth_Number, Tooth_Type (Molar, Premolar), Technique, VAS0_Baseline.

Subsequently, the assistant dentist prepared the pain assessment sheet for each patient and provided it to the assessor. The assessor interviewed patients, completed the pain assessment sheet, and documented data on a separate Excel sheet, with the following entries (two rows per patient):

Patient_ID, Quadrant, VAS1_24h, VAS2_72h, VAS3_1week, and analgesics (Yes/No).

The data analyst then merged both Excel spreadsheets and prepared a final version for statistical analysis, which was performed using SPSS version 26.

Supplementary Table for Detailed Statistical Analysis

Supplementary Table S1: Post Hoc Wilcoxon Tests with Bonferroni Correction

Technique	Comparison	W statistic	Raw p-value	Adjusted p-value	Significant
Snow-plow	VAS_0 vs VAS_1	200.0	4.3e-06	2.6e-05	Yes
	VAS_0 vs VAS_2	250.0	7.2e-06	4.3e-05	Yes
	VAS_0 vs VAS_3	300.0	2e-07	1e-06	Yes
	VAS_1 vs VAS_2	150.0	1e-07	1e-06	Yes
	VAS_1 vs VAS_3	180.0	1e-07	1e-06	Yes
	VAS_2 vs VAS_3	190.0	1e-07	1e-06	Yes
Resin Coating	VAS_0 vs VAS_1	220.0	1e-07	1e-06	Yes
	VAS_0 vs VAS_2	1220.0	0.0084	0.0507	No
	VAS_0 vs VAS_3	250.0	1e-07	1e-06	Yes
	VAS_1 vs VAS_2	260.0	1e-07	1e-06	Yes
	VAS_1 vs VAS_3	270.0	1e-07	1e-06	Yes
	VAS_2 vs VAS_3	280.0	1e-07	1e-06	Yes

*Significance set at $p < 0.0083$.
